# Nomogram for predicting rebleeding after initial endoscopic epinephrine injection monotherapy hemostasis in patients with peptic ulcer bleeding: a retrospective cohort study

**DOI:** 10.1186/s12876-022-02448-x

**Published:** 2022-07-31

**Authors:** Shan He, Linlin Liu, Liu Ouyang, Jingsong Wang, Nonghua Lv, Youxiang Chen, Xu Shu, Zhenhua Zhu

**Affiliations:** Department of Gastroenterology, The First Affiliated Hospital of Nanchang University, 17 Yongwaizheng, Street, Nanchang, 330006 Jiangxi Province China

**Keywords:** Epinephrine injection monotherapy, Rebleeding, Peptic ulcer bleeding, Risk factors, Nomogram

## Abstract

**Background:**

Although the current guidelines recommend endoscopic combination therapy, endoscopic epinephrine injection (EI) monotherapy is still a simple, common and effective modality for treating peptic ulcer bleeding (PUB). However, the rebleeding risk after EI monotherapy is still high, and identifying rebleeding patients after EI monotherapy is unclear, which is highly important in clinical practice. This study aimed to identify risk factors and constructed a predictive nomogram related to rebleeding after EI monotherapy.

**Methods:**

We consecutively and retrospectively analyzed 360 PUB patients who underwent EI monotherapy between March 2014 and July 2021 in our center. Then we identified independent risk factors associated with rebleeding after initial endoscopic EI monotherapy by multivariate logistic regression. A predictive nomogram was developed and validated based on the above predictors.

**Results:**

Among all PUB patients enrolled, 51 (14.2%) had recurrent hemorrhage within 30 days after endoscopic EI monotherapy. After multivariate logistic regression, shock [odds ratio (OR) = 12.691, 95% confidence interval (CI) 5.129–31.399, p < 0.001], Rockall score (OR = 1.877, 95% CI 1.250–2.820, p = 0.002), tachycardia (heart rate > 100 beats/min) (OR = 2.610, 95% CI 1.098–6.203, p = 0.030), prolonged prothrombin time (PT > 13 s) (OR = 2.387, 95% CI 1.019–5.588, p = 0.045) and gastric ulcer (OR = 2.258, 95% CI 1.003–5.084, p = 0.049) were associated with an increased risk of rebleeding after an initial EI monotherapy treatment. A nomogram incorporating these independent high-risk factors showed good discrimination, with an area under the receiver operating characteristic curve (AUROC) of 0.876 (95% CI 0.817–0.934) (p < 0.001).

**Conclusions:**

We developed a predictive nomogram of rebleeding after EI monotherapy, which had excellent prediction accuracy. This predictive nomogram can be conveniently used to identify low-risk rebleeding patients after EI monotherapy, allowing for decision-making in a clinical setting.

## Introduction

Peptic ulcer disease is defined as a break of the mucosal barrier that exposes the submucosa due to the damaging effects of acid and pepsin present in the gastroduodenal lumen [1, 2]. Peptic ulcer bleeding (PUB) is one of the most common and severe complications of peptic ulcer disease and accounts for the leading etiology of acute upper gastrointestinal bleeding (UGIB) [3, 4]. With the rapid improvement of endoscopic therapy and medication in recent years, many PUB patients can be treated without surgery. However, up to 10%-15% of PUB patients have recurrent bleeding after initial endoscopic hemostasis within 30 days, which greatly influences the prognosis of PUB [1–4]. Consequently, proper endoscopic hemostasis therapy should be taken into consideration depending on the characteristics of the patients.

Many authoritative guidelines, including the 2020 American Gastroenterological Association (AGA), 2021 American College of Gastroenterology (ACG) and 2021 European Society of Gastrointestinal Endoscopy (ESGE), recommend combination therapy using epinephrine injection plus a second hemostasis modality (contact thermal or mechanical therapy) for patients with actively bleeding ulcers (FIa, FIb) [5–7]. However, epinephrine injection (EI) monotherapy is widely used in real-world medical emergencies due to its low technical requirements, easy operation, low costs and quick hemostasis in primary hemostasis, especially in low-risk rebleeding PUB patients [5–11]. In addition, EI monotherapy in high-risk rebleeding patients in nontertiary hospitals can achieve temporary hemostasis, which will provide more referral time and safer referral opportunities. Therefore, it is vital to identify low-risk rebleeding patients suitable for EI monotherapy treatment, especially in nontertiary hospitals with no endoscopic combination therapy conditions and techniques. Early identification of high-risk patients may facilitate the choice of physician treatment modalities and the need for referral to tertiary hospitals. Therefore, the objective of this study was to identify risk factors related to rebleeding after initial EI monotherapy hemostasis for 30 days. Then, we developed and validated a nomogram that predicts the risk of rebleeding after EI monotherapy. Thus, additional combination therapy with EI is warranted in high-risk rebleeding patients to guarantee the safety and efficacy of endoscopic hemostasis.

## Methods

### Patients and data collection

This was a single-center and retrospective study. We consecutively enrolled peptic ulcer bleeding (PUB) patients who initially underwent epinephrine injection (EI) monotherapy for hemostasis at the First Affiliated Hospital of Nanchang University between March 2014 and July 2021. Data were collected from the endoscopy database and electronic medical record system of the First Affiliated Hospital of Nanchang University. Patients meeting the following criteria were excluded: (1) epinephrine injection combined with sclerosant or histoacryl injection therapies; (2) other hemostasis therapies, including mechanical (such as hemoclipping) or thermal (such as argon plasma coagulation (APC)) therapies; (3) patients diagnosed with other possible reasons for bleeding, such as esophageal and gastric varices, Dieulafoy lesions, malignant lesions, hemorrhagic erosive gastritis, esophageal foreign-body injury, or Mallory-Weiss syndrome, etc.; (4) patients with Forrest Ia peptic ulcers, which could not reach initial technical success for hemostasis after EI monotherapy; Forrest IIc and III peptic ulcers, which were not necessary for endoscopy intervention for hemostasis; and (5) patients with incomplete demographic data. Finally, a total of 360 patients were enrolled.

Patients’ basic characteristics were recorded at hospital admission. Data collection included demographic information (sex, age, smoking and drinking history, medication history, upper gastrointestinal bleeding history), comorbidity (strokes, coronary artery disease, chronic renal disease, liver cirrhosis, hypertension, diabetes), physical examinations (blood pressure, heart rate), laboratory findings, endoscopic findings (ulcer size, ulcer location and stigmata of hemorrhage), Glasgow Blatchford score, Rockall score, AIMS65 score and clinical outcomes. The study was approved by the Human Ethics Committee of The First Affiliated Hospital of Nanchang University. All patients provided written informed consent for the endoscopic procedure.

### Endoscopic evaluation and medication

**Fig. 1:**
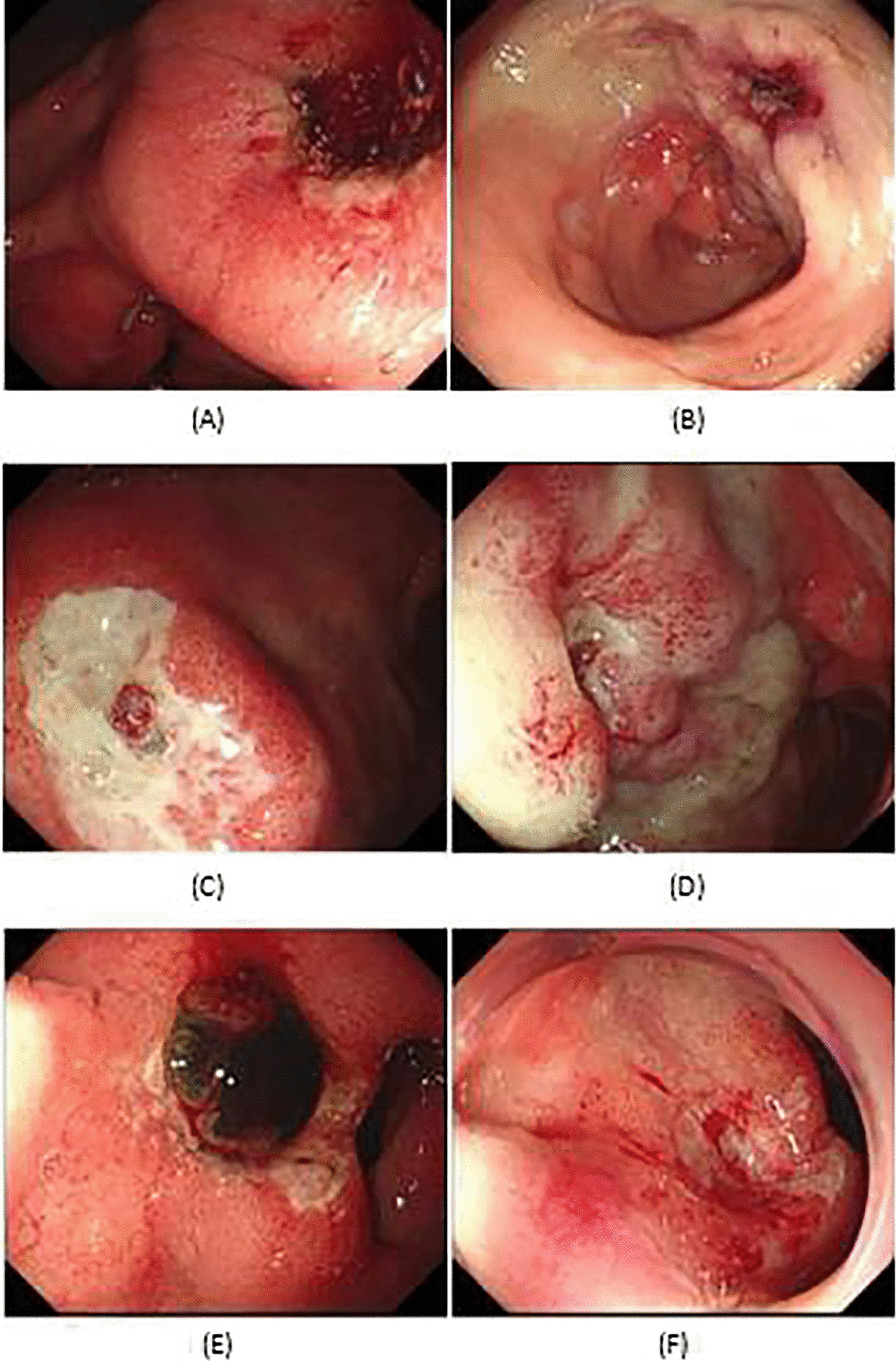
Endoscopic findings of standard epinephrine monotherapy in the treatment of PUB patients. **A** Forrest Ib PUB patients before EI therapy; **B** Forrest Ib PUB patients after EI therapy; **C** Forrest IIa PUB patients before EI therapy; **D** Forrest IIa PUB patients after EI therapy; **E** Forrest IIb PUB patients before EI therapy; **F** Forrest IIb PUB patients after EI therapy

In our center, all emergency endoscopic treatments were performed by experienced deputy directors or chief physicians within 24 h. Endoscopists were familiar with the indications, efficacy and limitations of currently available tools and techniques for endoscopic hemostasis and were comfortable applying endoscopic epinephrine injection therapies, they all have endoscopic experience of more than 5 years [5, 11]. In this study, we only chose those patients who underwent endoscopic EI monotherapy between March 2014 and July 2021 for enrollment. Diluted epinephrine (1:10,000 dilution, equivalent to 100 mcg/mL) can be injected at or near the bleeding site. All enrolled patients were injected the same volume of epinephrine injection and finally achieved technical hemostasis during the initial endoscopy. The bleeding status under endoscopy was classified based on the modified Forrest classification [12]. The endoscopic findings of standard epinephrine monotherapy in the treatment of PUB patients according to Forrest classification was displayed in Fig. 1. The most severe ulcer was used to classify patients with more than one ulcer. After endoscopy, all patients immediately received high-dose intravenous proton pump inhibitors (PPIs) (an 80-mg bolus injection followed by a continuous infusion of 8 mg per hour for 72 h). PPI included omeprazole, esomeprazole and pantoprazole. Then 40 mg PPI was taken orally once daily for 30 days after short-term (72 h) high-dose intravenous PPI therapy in the hospital. All patients were followed up for at least 30 days.

### Definition and outcome assessment

Rebleeding was defined as recurrent hematemesis, melena, anemia or vital hemodynamic instability with a decrease in hemoglobin by at least 2 g/dL after a successful initial endoscopic treatment within 30 days, and fresh blood could be seen in the stomach or duodenum during the second-look endoscopic observation [11, 13]. Patients who underwent a second endoscopic therapy for hemostasis within 30 days were also regarded as rebleeding. Shock was defned as shock index (pulse rate/systolic blood pressure) > 1.0 or systolic blood pressure < 90 mmHg. Technical success meant initial success for hemostasis during endoscopy. Clinical success meant hemostasis during endoscopy and no recurrent bleeding in 30 days follow-up.

The primary outcome of this study was to identify the main risk factors associated with rebleeding after initial endoscopy treatment, and we constructed and internally validated a novel predictive nomogram for rebleeding after initial therapy. Additionally, we calculated the rebleeding rate, time to rebleeding, need for surgery, the requirement for repeated endoscopic hemostasis and mortality.

### Statistical analysis

For normally distributed data, continuous variables are presented as the mean ± standard deviation (SD) and were analyzed by using Student’s t test. In contrast, continuous variables are presented as the median and interquartile range for abnormally distributed data. The Mann–Whitney U test was performed to analyze the data. Categorical variables were expressed as proportions, and the χ2 test or Fisher’s exact test was used to analyze the data as appropriate.

Variables associated with rebleeding (p < 0.05) were incorporated into the multivariate logistic regression analysis (backward stepwise) to identify the independent risk factors. All results are presented as odds ratios (ORs) and 95% confidence intervals (95% CIs). p < 0.05 was considered statistically significant. Then, a predictive nomogram was constructed based on the outcome of the final multivariate logistic regression analysis (p < 0.05). Receiver operating characteristic (ROC) curves were plotted to assess the predictive ability of the nomogram.

All statistical analyses were performed with IBM SPSS software version 24.0 for Windows (SPSS Inc., Chicago, IL, USA) and R statistical software 4.1.0 (www.r-project.org). A two-tailed p value < 0.05 was considered statistically significant.

## Results

### Baseline characteristics of enrolled patients

**Fig. 2:**
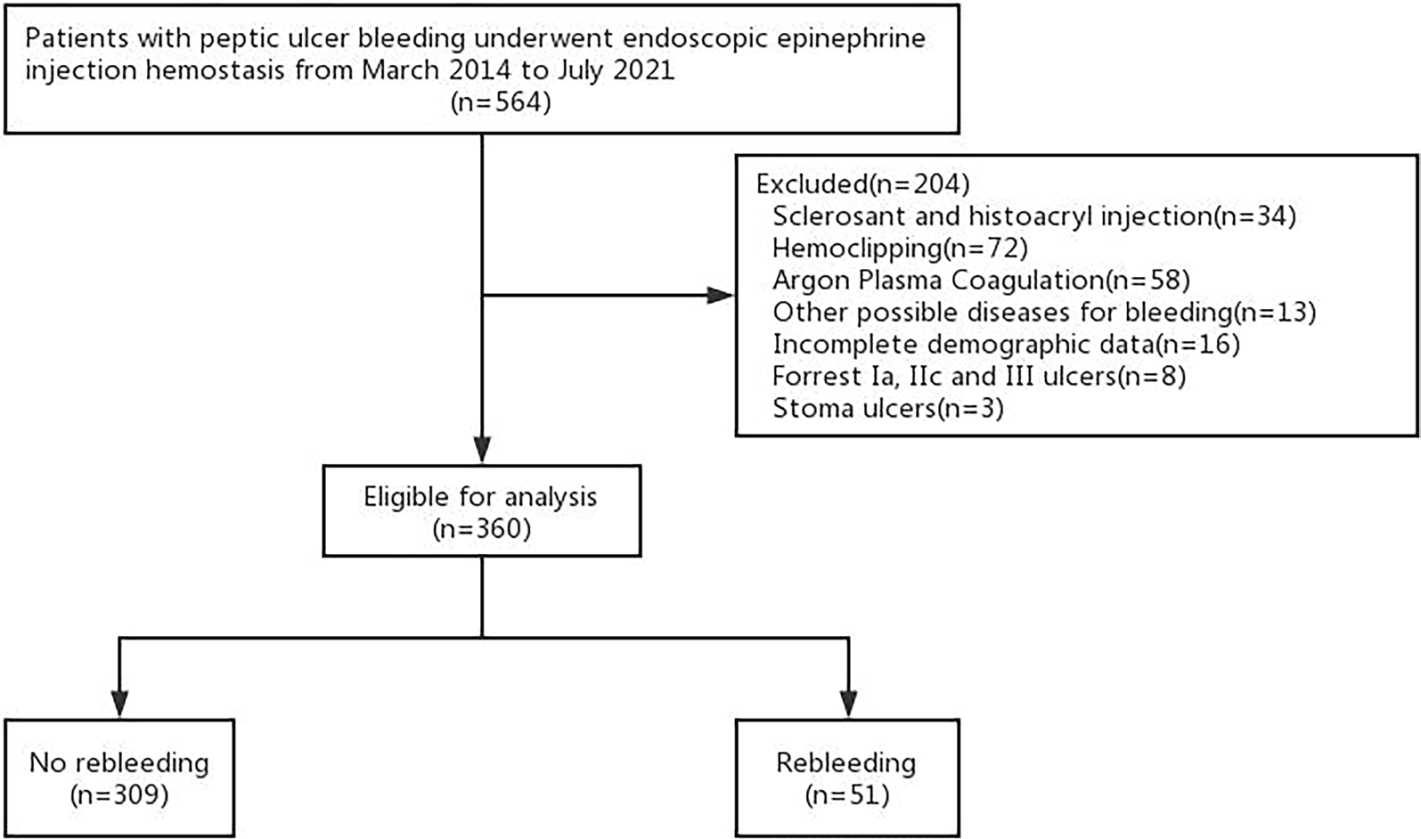
The Flowchart of patients included in this study

A total of 360 patients with acute peptic ulcer bleeding who met the inclusion criteria were finally enrolled in our study. They all underwent endoscopic epinephrine injection (EI) monotherapy for hemostasis and received at least a 30-day follow-up. After initial treatment, rebleeding occurred in 51 (14.2%) patients. Among the rebleeding group, the median time to rebleeding was 2 days; 20 (39.2%) patients underwent surgery, 13 (25.5%) patients died due to rebleeding, and 18 (35.3%) patients received repeat endoscopic therapy (Fig. 2, Table 4).

The baseline characteristics of the enrolled patients are presented in Tables 1, 2 and 3. Among the 360 enrolled patients, the median age was 50 (IQR, 37–61) years old, up to 105 (29.2%) cases were more than 60 years old, and most (83.9%) were male patients. Additionally, the most common site for peptic ulcer bleeding was the duodenum (71.7%), followed by the stomach (28.3%), with at least 12.2% of patients having a large ulcer size (> 20 mm). These patients had a median systolic blood pressure of 116 (IQR, 105–128) mmHg; only 20 (5.6%) patients had low blood pressure (systolic blood pressure < 90 mmHg), but up to 11.9% of them bled to shock (shock index > 1), mostly (58.8%) in the rebleeding groups. For laboratory findings, the median hemoglobin (HB) level on admission was 87 (IQR, 72–110.8) g/L, and albumin (ALB) was 35.9 (30.9–40.2) g/L. A prolonged prothrombin time (PT > 13 s) was observed in 68 patients (18.9%), while up to 45.1% of them were in the rebleeding group. The comorbidities included hypertension, type 2 diabetes mellitus, strokes, coronary artery disease, liver cirrhosis, cancer and renal failure, with hypertension (24.7%) being the most frequent comorbidity. Furthermore, only 18 (5.0%) patients were antiplatelet drug users, 11 (3.1%) patients were taking nonsteroidal anti-inflammatory drugs (NSAIDs), and only 6 (1.7%) were anticoagulant users. A total of 102 (28.3%) patients were smokers, and 62 (17.2%) patients were addicted to alcohol (Table 4).

**Table 1:** Clinical characteristics between non-rebleeding and rebleeding groups

Variables	All subjects (N = 360)	Non-rebleeding (N = 309)	Rebleeding (N = 51)	*p* value
Sex (n [%])				0.463
Male	302 (83.9)	261 (84.5)	41 (80.4)	
Female	58 (16.1)	48 (15.5)	10 (19.6)	
Age(years), [median (IQR)]	50 (37–61)	50 (37–60.5)	55 (36–65)	0.226
Age ≥ 60 years (n [%])	105 (29.2)	86 (27.8)	19 (37.3)	0.172
Alcoholics (n [%])	62 (17.2)	55 (17.8)	7 (13.7)	0.475
Smokers (n [%])	102 (28.3)	87 (28.2)	15 (29.4)	0.854
Medication history (n[%])				
Use of anti-platelets	18 (5.0)	14 (4.5)	2 (3.9)	0.845
Use of NSAIDS	11 (3.1)	9 (2.9)	2 (3.9)	0.698
Use of anticoagulants	6 (1.7)	6 (1.9)	0 (0)	1
PUB history (n [%])	96 (26.7)	81 (26.2)	15 (29.4)	0.632
Comorbidity (n [%])				
Strokes	10 (2.8)	6 (1.9)	4 (7.8)	0.018
Coronary artery disease	7 (1.9)	7 (2.3)	0 (0)	0.278
Chronic renal disease	24 (6.7)	20 (6.5)	4 (7.8)	0.716
Liver cirrhosis	11 (3.1)	7 (2.3)	4 (7.8)	0.032
Hypertension	89 (24.7)	75 (24.3)	14 (27.5)	0.626
Diabetes mellitus	28 (7.8)	25 [8.1]	3 (5.9)	0.585
Systolic blood pressure(mm Hg), [median(IQR)]	116 (105–128)	117 (106–128)	115 (103–130)	0.824
Systolic blood pressure < 90 mmHg (n [%])	20 (5.6)	18 (5.8)	2 (3.9)	0.582
Heart rate (beats/min), [median (IQR)]	85 (75–96)	84 (75–94)	92 (74–106)	0.018
Heart rate > 100 beats/min (n [%])	60 (16.7)	39 (12.6)	21 (41.2)	< 0.001
Shock (n [%])	43 (11.9)	13 (4.2)	30 (58.8)	< 0.001
Glasgow Blatchford Score, median (IQR)	9 (7–11)	9 (7–11)	12 (9–14)	< 0.001
Rockall Score, median (IQR)	4 (3–5)	4 (3–4.5)	5 (4–6)	< 0.001
AIMS65 Score, median (IQR)	0 (0–1)	0 (0–1)	1 (1–2)	< 0.001

NSAIDS, Non-Steroidal Anti-Inflammatory Drugs; PUB, peptic ulcer bleeding; IQR, interquartile range

**Table 2:** Laboratory findings between non-rebleeding and rebleeding groups

Variables	All subjects (N = 360)	Non-rebleeding (N = 309)	Rebleeding (N = 51)	*p* value
HB on admission (g/L), median(IQR)	87 (72–110.8)	87 (73–114)	76 (62–97)	0.004
HB < 100 g/L (n [%])	234 [65.0]	195 [63.1]	39 [76.5]	0.064
WBC (× 10^9^/L), median(IQR)	8.9 (6.5–11.8)	8.8 (6.4–11.7)	9.2 (7.5–14.3)	0.129
PLT (× 10^9^/L), median (IQR)	191 (147.3–239.8)	191 (149.5–239.5)	189 (127–255)	0.664
BUN (mmol/L), median(IQR)	9.5 (6.5–13.0)	9.4 (6.4–12.6)	10.1 (6.9–13.8)	0.151
Cr (μmol/L), median (IQR)	73.5 (60.9–86.5)	73.3 (60.8–86.5)	74.4 (61.3–87.2)	0.766
ALB (g/L), median (IQR)	35.9 (30.9–40.2)	36.4 (31.6–40.8)	32.3 (27.1–36.7)	< 0.001
ALB < 30 g/L (n [%])	74 (20.6)	51 (16.5)	23 (45.1)	< 0.001
PT(s), median (IQR)	11.7 (11.1–12.6)	11.7 (11.1–12.5)	12.3 (11.6–14.9)	0.001
PT > 13 s (n [%])	68 (18.9)	45 (14.6)	23 (45.1)	< 0.001
APTT(s), median (IQR)	24.3 (22.2–28.3)	24.1 (22.2–27.7)	26.8 (23.4–33.2)	0.005
INR, median (IQR)	1.03 (0.98–1.11)	1.03 (0.98–1.1)	1.07 (1–1.25)	0.009

HB, Hemoglobin; WBC, White Blood Cell Count; PLT, Platelet; BUN, Blood Urea Nitrogen; Cr, Creatinine; ALB, Albumin; PT, Prothrombin Time; APTT, Activated Partial Thromboplastin Time; IQR, interquatile range; INR, International Normalized Ratio

**Table 3:** Endoscopic findings between non-rebleeding and rebleeding groups

Variables	All subjects (N = 360)	Non-rebleeding (N = 309)	Rebleeding (N = 51)	*p* value
Ulcer size (mm), median (IQR)	8 (5–12)	8 (5–10)	10 (8–15)	0.001
Ulcer size > 20 mm (n [%])	44 (12.2)	29 (9.4)	15 (29.4)	< 0.001
Ulcer location (n [%])				0.004
Duodenum	258 (71.7)	230 (74.4)	28 (54.9)	
Stomach	102 (28.3)	79 (25.6)	23 (45.1)	
Stigmata of hemorrhage (n [%])				0.026
Forrest Ib	105 (29.2)	83 (26.9)	22 (43.1)	
Forrest IIa	118 (32.8)	101 (32.7)	17 (33.3)	
Forrest IIb	137 (38.0)	125 (40.4)	12 (23.6)	

IQR, interquartile range

**Table 4:** Outcome of rebleeding groups

Variables	N = 51
Time to rebleeding, median (IQR), days	2 (1–3)
Need for surgery (n [%])	20 (39.2)
Repeated endoscopic hemostasis (n [%])	18 (35.3)
Mortality (n [%])	13 (25.5)

IQR, interquartile range

### Comparison between the nonrebleeding and rebleeding groups

Compared with the nonrebleeding group, the rebleeding group showed significantly higher rates of shock (58.8% vs. 4.2%; p < 0.001) and tachycardia (heart rate > 100 beats/min) (41.2% vs. 12.6%; p < 0.001). Patients who suffered from stroke (7.8% vs. 1.9%; p = 0.018) or liver cirrhosis (7.8% vs. 2.3%; p = 0.032) were more susceptible to rebleeding after initial epinephrine monotherapy. Rebleeding was also closely associated with the Glasgow Blatchford score (p < 0.001), Rockall score (p < 0.001) and AIMS65 score (p < 0.001) (Table 1). For laboratory findings, rebleeding patients had a significantly lower HB (76 (IQR, 62–97) vs. 87 (IQR, 73–114); P = 0.004) and ALB (32.3 (IQR, 27.1–36.7) vs. 36.4 (IQR, 31.6–40.8); P < 0.001); but a higher prothrombin time (PT) (12.3 (IQR, 11.6–14.9) vs. 11.7 (IQR, 11.1–12.5); p = 0.001), activated partial thromboplastin time (APTT) (26.8 (IQR, 23.4–33.2) vs. 24.1 (IQR, 22.2–27.7); p = 0.005) and international normalized ratio (INR) (1.07 (IQR, 1–1.25) vs. 1.03 (IQR, 0.98–1.1); p = 0.009) (Table 2). Additionally, the ulcer size (p = 0.001) and location (p = 0.004) were significantly different, with a large ulcer size (> 20 mm) (29.4% vs. 9.4%) and gastric ulcer (45.1% vs. 25.6%) being more frequent in the rebleeding groups. Peptic ulcers with high-risk stigmata (FIb and FIIa) had a remarkably higher rebleeding risk (43.1% vs. 26.9%). Patients whose endoscopic performance revealed an adherent clot (FIIb) did not manifest a higher rebleeding rate (23.6% vs. 40.4%) (Table 3).

### Multivariate analysis of risk factors for rebleeding after initial treatment

Risk factors significantly influencing rebleeding (p < 0.05) after an initial successful technical treatment were analyzed by multivariate logistic regression analysis by backward stepwise regression (Table 5). Shock [odds ratio (OR) = 12.691, 95% confidence interval (CI) 5.129–31.399, p < 0.001], Rockall score (OR = 1.877, 95% CI 1.250–2.820, p = 0.002), tachycardia (heart rate > 100 beats/min) (OR = 2.610, 95% CI 1.098–6.203, p = 0.030), prolonged prothrombin time (PT > 13 s) (OR = 2.387, 95% CI 1.019–5.588, p = 0.045) and gastric ulcer (OR = 2.258, 95% CI 1.003–5.084, p = 0.049) were associated with an increased risk of rebleeding after an initial EI monotherapy treatment, while other factors were not included in the model. All these risk predictors remained statistically significant after multivariate adjustment.

**Table 5:** Risk factors associated with recurrent bleeding after EI monotherapy by multivariate logistic regression (backward stepwise)

Variables	B	SE	Wald	OR (95%CI)	p value
Shock	2.541	0.462	30.221	12.691 (5.129–31.399)	< 0.001
Rockall Score	0.630	0.208	9.202	1.877 (1.250–2.820)	0.002
Heart rate > 100	0.959	0.442	4.719	2.610 (1.098–6.203)	0.030
PT > 13 s	0.870	0.434	4.018	2.387 (1.019–5.588)	0.045
Gastric ulcer	0.815	0.414	3.872	2.258 (1.003–5.084)	0.049
Constant	-5.935	0.960	38.207	0.003	< 0.001

OR, odds ratio; CI, confidence interval

### Predictive nomogram and performance of the models

**Fig. 3:**
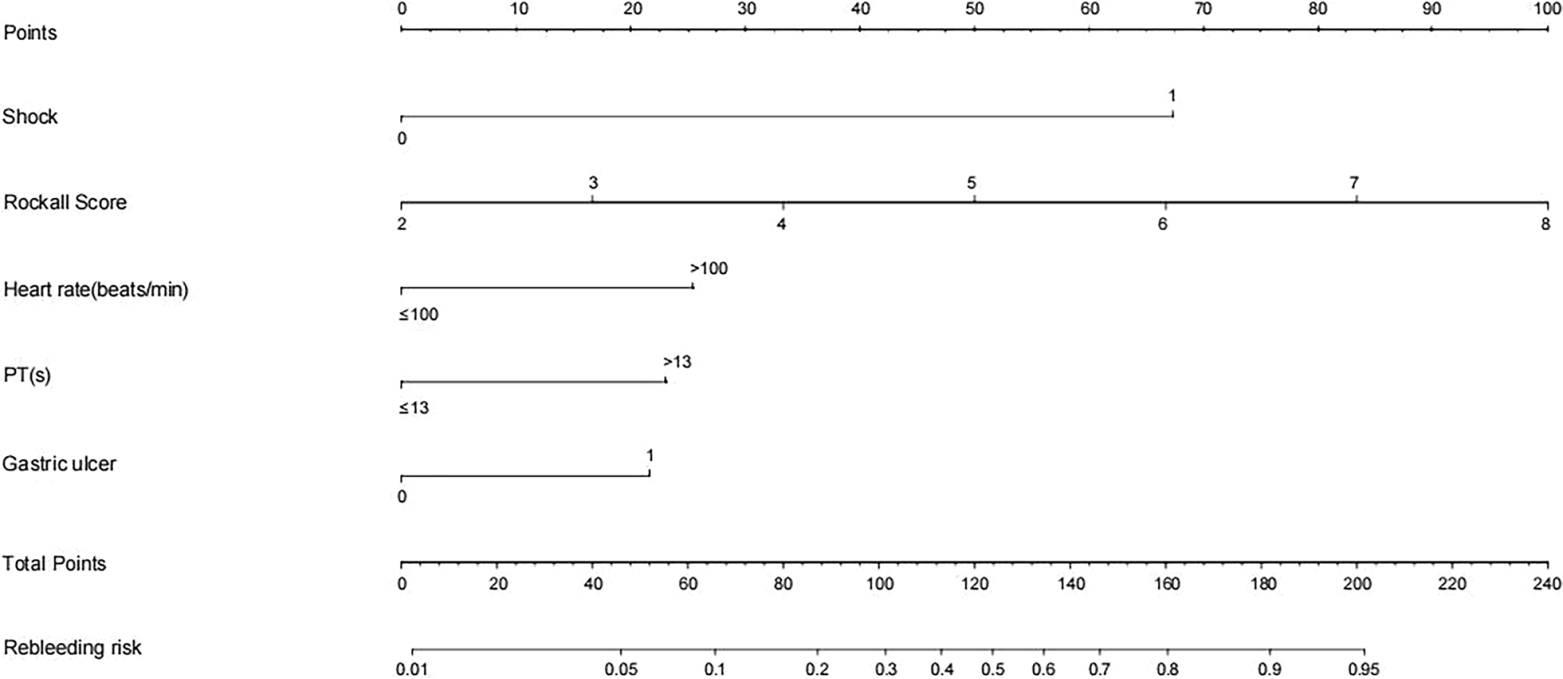
Predictive nomogram for recurrent bleeding after single endoscopic epinephrine injection therapy

A predictive nomogram of rebleeding after the initial EI monotherapy was constructed based on the multivariate logistic regression analysis results (Fig. 3). The model assigned a weighted point value to each independent risk factor on the scale. A higher total score for all risk factors was associated with a higher risk of rebleeding.

**Fig. 4:**
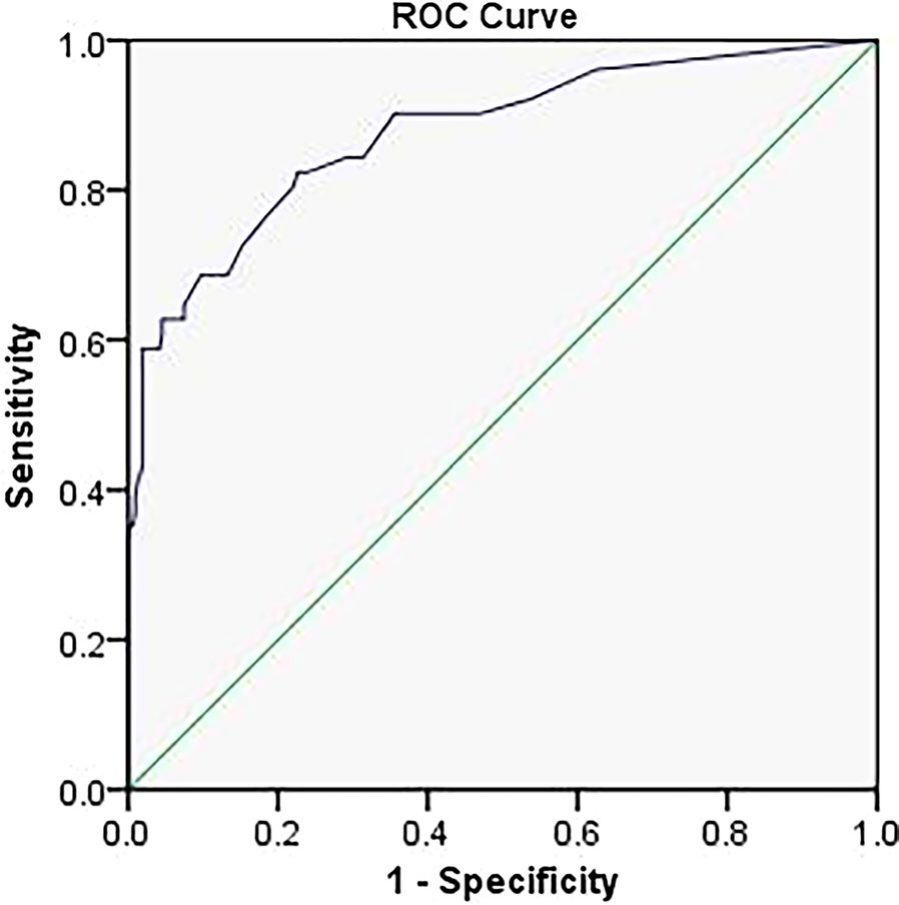
ROC Curve showing the predictive ability for Rebleeding

**Fig. 5:**
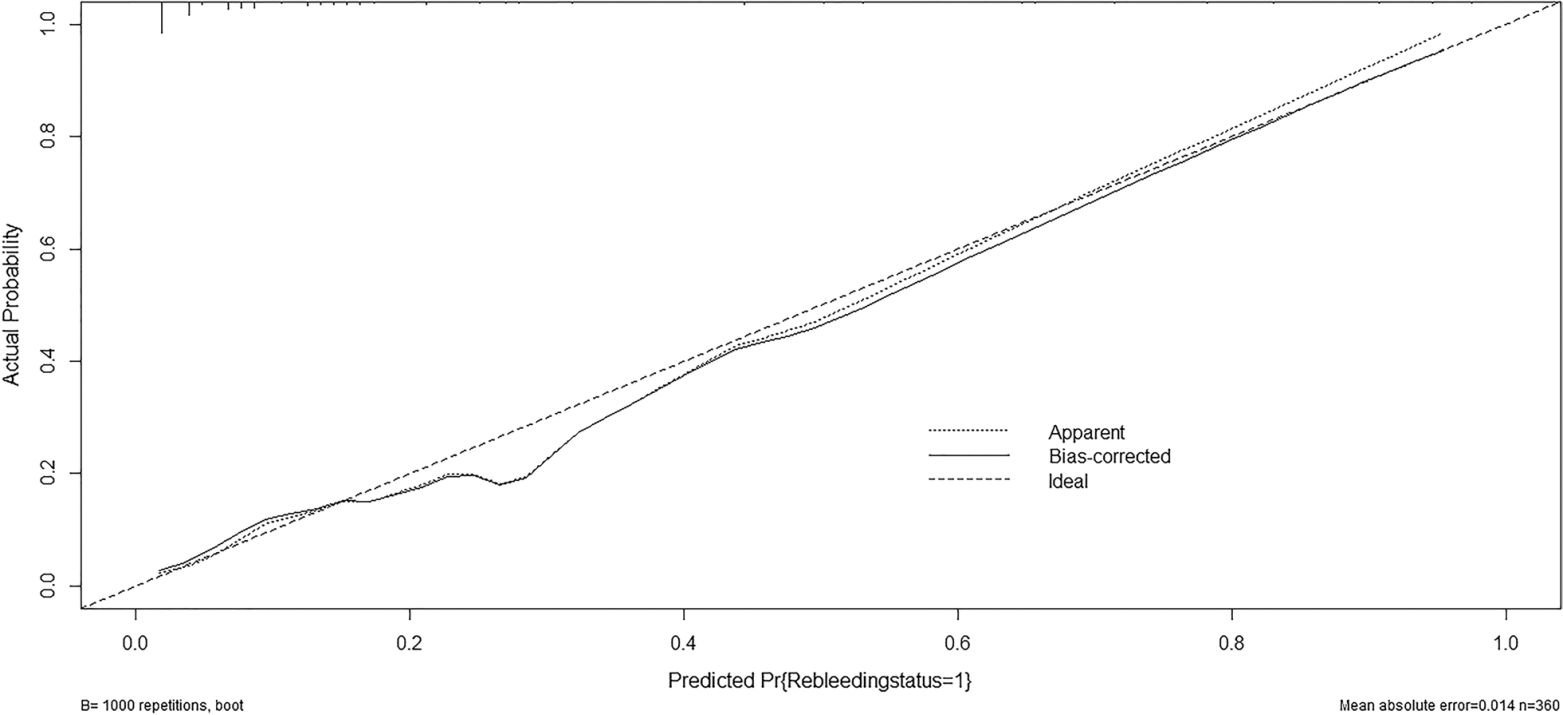
Calibration curves of the nomogram. “Rebleeding status = 1” means “Rebleeding”, “Pr” means “Probability”

The predictive ability of the nomogram was analyzed by receiver operating characteristic (ROC) curve analysis (Fig. 4) and internally validated via bootstrapping resampling of the construction data set (with 1000 bootstrap samples per model) to obtain optimism corrected discrimination via the C-index for rebleeding. The calibration curve (Fig. 5) of the nomogram showed a good fit between the prediction and observation in the primary cohort. The area under the ROC curve (AUC) was 0.876 (95% CI 0.817–0.934) (p < 0.001), with a sensitivity of 82.40% and a specificity of 77.30% (Table 6), indicating that the sum risk score had high accuracy for the prediction of rebleeding risk after the initial treatment.

**Table 6:** Predictive ability of the model

	Sensitivity	Specificity	AUC	95% CI	P
Predictive model	82.40%	77.30%	0.876	0.817–0.934	< 0.001

CI, confidence interval

## Discussion

Rebleeding often occurs within 30 days of EI administration with a high incidence after initial endoscopic hemostasis, which is closely associated with severe complications and high mortality. Meanwhile, a high rebleeding rate can negatively impact the patient’s quality of life and result in a significant financial burden [4, 11, 13]. Therefore, the most important objective of endoscopic treatment is to achieve persistent hemostasis and minimize the rebleeding risk after endoscopic treatment. Concerning PUB with high-risk stigmata (active bleeding or visible vessels), a second hemostasis modality (such as thermal, mechanical or sclerosant injection) in combination with EI can significantly reduce the morbidity and mortality of rebleeding, which is strongly recommended in most guidelines and clinical trials [5–7, 14]. Recent meta-analyses show EI monotherapy is inferior to EI combination therapy (EI plus thermal or mechanical therapy) in terms of rebleeding and the need for emergency surgery postendoscopy [15, 16]. However, EI monotherapy tends to be widely used among patients because of its accessibility and low expenditure, especially in nontertiary hospitals. EI monotherapy also has high efficacy in hemostasis and is easy to operate during emergencies [5–10]. Therefore, identifying risk factors for rebleeding after EI monotherapy can help guide our management of PUB patients during endoscopy.

**Fig. 6:**
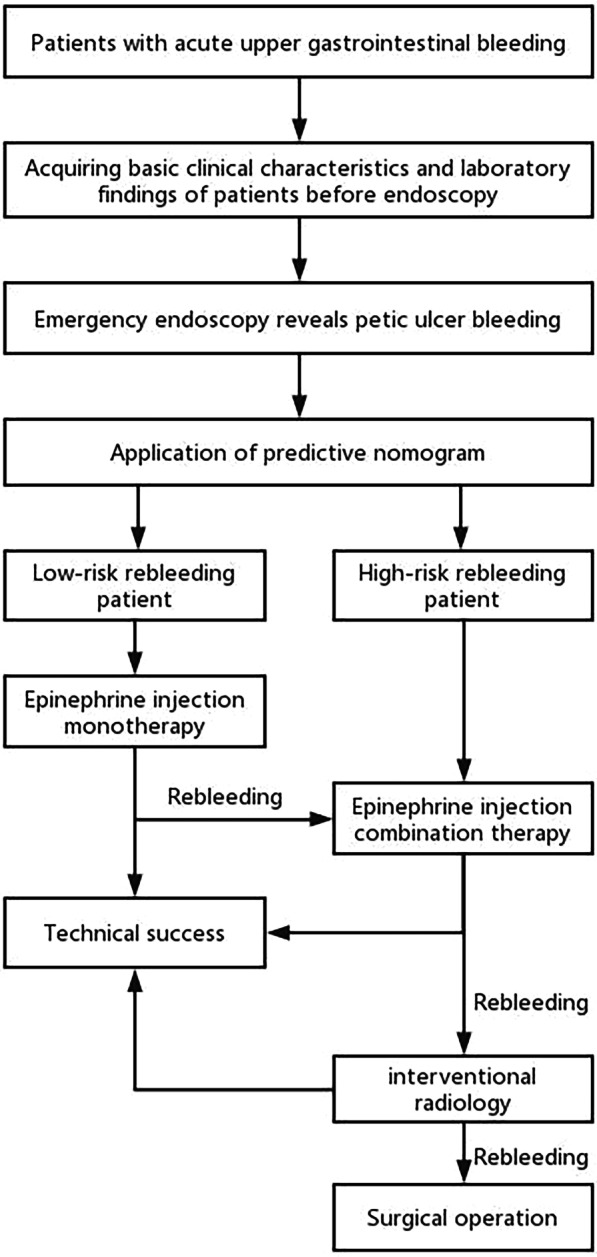
Algorithm for the management of peptic ulcer bleeding patients according to the predictive Nomogram

In this study, we analyzed the risk factors associated with rebleeding after EI monotherapy based on a detailed database of PUB patients in our hospital. After multivariate logistic regression analysis, we found that shock, tachycardia (heart rate > 100 beats/min), gastric ulcer bleeding, a higher Rockall score and a prolonged prothrombin time (PT > 13 s) were independently associated with a high rebleeding rate after EI monotherapy. Additionally, we developed an effective predictive model and nomogram for rebleeding after EI monotherapy based on these risk factors with sound discrimination. After that, we proposed an algorithm for the management of PUB patients (Fig. 6). When we encounter patients with acute gastrointestinal bleeding, we first acquire basic clinical characteristics and laboratory findings before endoscopy, including age, blood pressure, heart rate, hemoglobin, albumin, prothrombin time (PT), underlying diseases, etc. After proper triage, medical management and stabilization, all patients underwent emergency endoscopy within 24 h. If endoscopy revealed peptic ulcer bleeding (PUB), we performed assessments for shock, heart rate, gastric ulcer bleeding, Rockall score and PT to be evaluated by the predictive nomogram. If the patient is considered a low-risk rebleeding patient, we can initially employ EI monotherapy for hemostasis. Conversely, if the patient is classified into a high-risk rebleeding population, a combination therapy rather than EI monotherapy is more suggested for hemostasis. Furthermore, if hemorrhage recurs within 30 days after the initial combination therapy, interventional radiology, such as transcatheter angiographic embolization (TAE), should be considered following combination therapy. Finally, surgery is indicated after failed TAE [5–7, 11, 13].

In this study, we found that patients with shock, a prolonged prothrombin time and tachycardia were more likely to have a recurrent hemorrhage in the future, which was consistent with some previous similar studies [17–19]. Thomopolous et al. [18] proposed that shock, use of NSAIDs and history of ulcer bleeding were independent risk factors for rebleeding after EI. Park et al. [19] built a scoring system for predicting the probability of a patient with acute upper gastrointestinal bleeding requiring an operation, indicating that patients with an elevated heart rate, a high BMI and peptic ulcer in the lesser gastric curve were closely related to a high rebleeding rate after EI therapy. We think that shock and tachycardia on admission may result from persistent blood loss, which will cause the loss of platelets and coagulation factors. In addition, mass rehydration to correct hemodynamic instability leading to hemodilution further aggravates coagulation dysfunction with prolonged prothrombin time, which poses a great threat to persistent hemostasis after initial technical success of EI monotherapy. Unlike other clinical trials and guidelines [20, 21], our present study showed no significant differences in rebleeding in the use of NSAID (3.9% vs. 2.9%; p = 0.698) or antiplatelet (3.9% vs. 4.5%; p = 0.845) drugs; we think this may be due to the small sample size of the chosen groups. The use of the Glasgow Blatchford score and Rockall score in the prediction of rebleeding in PUB patients after endoscopy EI remains controversial in different studies [22, 23]. Budimir et al. [22] reported that the Glasgow Blatchford score better predicted overall rebleeding risk than the Rockall score. However, Liu et al. [23] concluded that the Rockall and Glasgow Blatchford scores are equivalent in predicting the rebleeding of acute upper gastrointestinal bleeding. In our study, the Rockall score was an independent predictor of rebleeding, but the Glasgow Blatchford score still had a high efficacy (p < 0.001) in predicting rebleeding. Therefore, the two scores are always performed together in the clinical use of prediction of rebleeding after endoscopic treatment. However, 25.5% (13/51) patients died due to rebleeding, which was little higher than some previous studies [24, 25]. Over half (7/13, 53.8%) of the dead population were combined with serious underlying diseases such as hepatic and renal failure in our rebleeding group, which we think was the main factor leading to the high mortality in our study. Sometimes, some factors such as incomplete endoscopic hemostasis at initial EI monotherapy, bad transfer to additional surgery or interventional radiology, and missing adequate timing for transfusion could also accelerate rebleeding and thus lead to death.

The limitations of this study may include the following aspects. Firstly, this is a single-center retrospective study, which could introduce selection bias due to the nature of retrospective study. Further multicenter and prospective clinical trials with large samples are still warranted to validate the findings in the future. Secondly, EI therapy was performed by various levels of endoscopists, which might result in subtle differences in prognosis. Finally, due to the small sample size of rebleeding groups, we didn’t have a validation data set initially. However, we have internally validated the accuracy of our model by performing ROC curves. The area under the ROC curve (AUC) was 0.876 (95% CI 0.817–0.934) (p < 0.001), with a sensitivity of 82.40% and a specificity of 77.30%, which had a high accuracy and indeed helped us identify enough high-risk rebleeding patients in our clinical practice. Nonetheless, to our knowledge, the present study is the first to construct a nomogram to prevent rebleeding after EI monotherapy of PUB patients. This predictive model could help provide clinicians with an intuitive and quantitative tool for predicting rebleeding risk after EI monotherapy, which may be practical for clinicians to choose suitable modalities for PUB hemostasis.

However, the risk assessment of the patients should not influence the choice of endoscopic treatment modality of PUB. After hemostasis by EI monotherapy, EI combination therapy should be used regardless of the patient's rebleeding risk and physical condition. The modality of definitive hemostasis such as thermal, mechanical or sclerosant injection should be used depending on the availability of the method, familiarity with the method, the skill of the operator whenever it is possible.

## Conclusions

In conclusion, we developed a predictive nomogram of rebleeding after EI monotherapy, with excellent prediction accuracy and discriminatory ability. This predictive nomogram can be conveniently used to identify low-risk rebleeding patients after EI monotherapy, allowing for decision-making in a clinical setting. Nevertheless, EI monotherapy should be avoided in high-risk rebleeding patients, particularly in patients with shock, tachycardia (heart rate > 100 beats/min), gastric ulcer bleeding, a higher Rockall score and a prolonged prothrombin time (PT > 13 s). EI combined with thermal, sclerosant injection, or mechanical (such as clips) hemostasis is the initiative to treat these high-risk patients.

## Data Availability

The datasets used and/or analysed during the current study are available from the corresponding author on reasonable request. The data that support the findings of this study are available from the The First Affiliated Hospital of Nanchang University, but restrictions apply to the availability of these data, which were used under license for the current study, and so are not publicly available. Data are however available from the authors upon reasonable request and with permission of The First Affiliated Hospital of Nanchang University.

## Abbreviations

EI: Endoscopic epinephrine injection
UGIB: Upper gastrointestinal bleeding
PUB: Peptic ulcer bleeding
PPIs: Proton pump inhibitors
APC: Argon plasma coagulation
HB: Hemoglobin
WBC: White Blood Cell Count
PLT: Platelet
BUN: Blood Urea Nitrogen
Cr: Creatinine
ALB: Albumin
PT: Prothrombin Time
APTT: Activated Partial Thromboplastin Time
IQR: Interquatile range
INR: International Normalized Ratio
AUC: Area under curve
INR: International normalized ratio
ROC: Receiver operating characteristic
CI: Confdence interval
OR: Odds ratio
SD: Standard deviation
